# Quantifying the utility of islet autoantibody levels in the prediction of type 1 diabetes in children

**DOI:** 10.1007/s00125-022-05799-y

**Published:** 2022-10-05

**Authors:** Kenney Ng, Vibha Anand, Harry Stavropoulos, Riitta Veijola, Jorma Toppari, Marlena Maziarz, Markus Lundgren, Kathy Waugh, Brigitte I. Frohnert, Frank Martin, Olivia Lou, William Hagopian, Peter Achenbach, for the T1DI Study Group

**Affiliations:** 1 IBM Research, Cambridge, MA USA; 2 IBM Research, Yorktown Heights, NY USA; 3 Department of Pediatrics, PEDEGO Research Unit, University of Oulu and Oulu University Hospital, Oulu, Finland; 4 Institute of Biomedicine and Centre for Population Health Research, University of Turku, Turku, Finland; 5 Department of Pediatrics, Turku University Hospital, Turku, Finland; 6 Department of Clinical Sciences Malmö, Lund University, Malmö, Sweden; 7 Department of Pediatrics, Kristianstad Hospital, Kristianstad, Sweden; 8 Barbara Davis Center for Diabetes, University of Colorado, Denver, CO USA; 9 JDRF International, New York, NY USA; 10 Pacific Northwest Research Institute, Seattle, WA USA; 11 Institute of Diabetes Research, Helmholtz Zentrum München, German Research Center for Environmental Health, Munich-Neuherberg, Germany

**Keywords:** Islet autoantibody levels, Machine learning, Risk prediction models, Type 1 diabetes

## Abstract

**Aims/hypothesis:**

The aim of this study was to explore the utility of islet autoantibody (IAb) levels for the prediction of type 1 diabetes in autoantibody-positive children.

**Methods:**

Prospective cohort studies in Finland, Germany, Sweden and the USA followed 24,662 children at increased genetic or familial risk of developing islet autoimmunity and diabetes. For the 1403 who developed IAbs (523 of whom developed diabetes), levels of autoantibodies against insulin (IAA), glutamic acid decarboxylase (GADA) and insulinoma-associated antigen-2 (IA-2A) were harmonised for analysis. Diabetes prediction models using multivariate logistic regression with inverse probability censored weighting (IPCW) were trained using 10-fold cross-validation. Discriminative power for disease was estimated using the IPCW concordance index (C index) with 95% CI estimated via bootstrap.

**Results:**

A baseline model with covariates for data source, sex, diabetes family history, HLA risk group and age at seroconversion with a 10-year follow-up period yielded a C index of 0.61 (95% CI 0.58, 0.63). The performance improved after adding the IAb positivity status for IAA, GADA and IA-2A at seroconversion: C index 0.72 (95% CI 0.71, 0.74). Using the IAb levels instead of positivity indicators resulted in even better performance: C index 0.76 (95% CI 0.74, 0.77). The predictive power was maintained when using the IAb levels alone: C index 0.76 (95% CI 0.75, 0.76). The prediction was better for shorter follow-up periods, with a C index of 0.82 (95% CI 0.81, 0.83) at 2 years, and remained reasonable for longer follow-up periods, with a C index of 0.76 (95% CI 0.75, 0.76) at 11 years. Inclusion of the results of a third IAb test added to the predictive power, and a suitable interval between seroconversion and the third test was approximately 1.5 years, with a C index of 0.78 (95% CI 0.77, 0.78) at 10 years follow-up.

**Conclusions/interpretation:**

Consideration of quantitative patterns of IAb levels improved the predictive power for type 1 diabetes in IAb-positive children beyond qualitative IAb positivity status.

**Graphical abstract:**

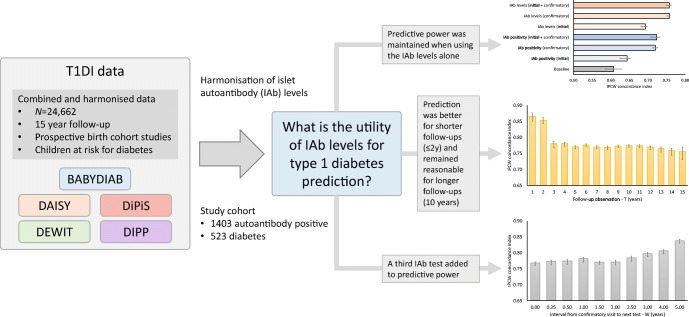

**Supplementary Information:**

The online version contains peer-reviewed but unedited supplementary material available at 10.1007/s00125-022-05799-y.

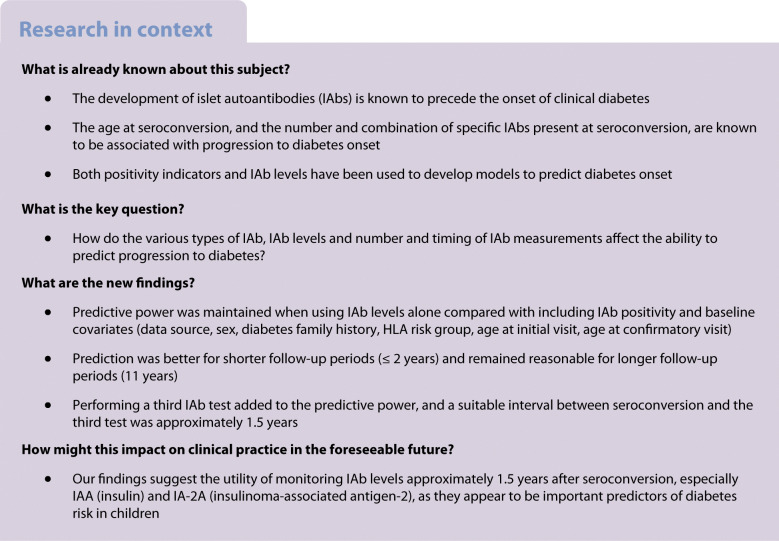



## Introduction

Accurate prediction of the onset of type 1 diabetes in children is important. It can usefully inform population screening, monitoring for metabolic instability, recruitment for clinical trials and timing of potential therapies [1]. The development of islet autoantibodies (IAbs) is known to precede the onset of clinical diabetes. However, the rate of progression from seroconversion to diabetes is highly heterogeneous. The age at seroconversion, and the number and combination of specific IAbs present at seroconversion, are known to be associated with progression to onset of diabetes [2–4]. Both positivity indicators and IAb levels have been shown to be associated with progression to diabetes [5–9], and both have also been used to develop models to predict diabetes onset [10–16].

In our previous work [17], we harmonised IAb levels from our large, prospective Type 1 Diabetes Intelligence (T1DI) study cohort [18], identified the IAb type-specific titre thresholds (measured at the time of confirmed positivity) that maximised discrimination of 5-year type 1 diabetes risk, and used the thresholds to risk-stratify children in various age groups via survival analysis. This prior work demonstrated that IAb levels were useful in predicting type 1 diabetes onset, and motivated us to perform a more comprehensive assessment of the utility of measurement of IAb levels. Specifically, we wished to evaluate how well progression to diabetes can be predicted and characterised by IAb information, i.e. which IAb types and IAb levels (as continuous variables) are useful for predicting rapid vs slow progression, and how the number and timing of IAb measurements affect robust prediction. We focused our analyses around the time point of seroconversion, defined as the time at which IAb positivity that was confirmed in a second consecutive sample first appeared. In this study, we built prediction models and used the harmonised IAb levels from our T1DI study cohort to investigate (1) how well IAb information at seroconversion predicts future diabetes onset; (2) how prediction performance changes as the follow-up observation period increases; and (3) the predictive value of IAb information measured at various times after seroconversion.

## Methods

### Study population

**Fig. 1:**
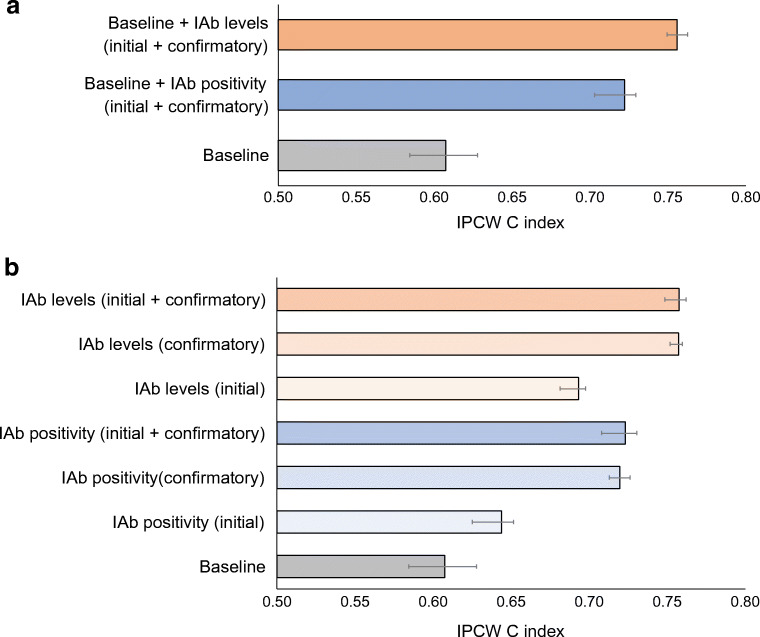
Type 1 diabetes prediction performance (IPCW concordance index [C index] with 95% CI) for various covariate sets. (**a**) Performance for a model using baseline covariates; a model using baseline covariates and IAb positivity indicators from both initial and confirmatory visits; and a model using baseline covariates and IAb levels from both initial and confirmatory visits. (**b**) Performance for a model using baseline covariates, models using IAb positivity indicators from the initial visit, the confirmatory visit and both visits, and models using IAb levels from the initial visit, the confirmatory visit and both visits. The prediction start time (‘time 0’) was the seroconversion confirmatory visit. The duration of the follow-up period was 10 years. IAbs include GADA, IA-2A and IAA

Prospective studies in Finland (DIPP [19]), Germany (BABYDIAB [20]), Sweden (DiPiS [21]) and the USA (DAISY [22] and DEW-IT [23]) have followed 24,662 children at increased genetic and familial risk of development of IAbs and diabetes, from close to birth for a period of 15 years, or until diagnosis. Data from these studies were combined and harmonised in the T1DI study cohort [18]. Only those children who seroconverted to autoantibodies against insulin (IAA), glutamic acid decarboxylase (GADA) or insulinoma-associated antigen-2 (IA-2A), with autoantibody level measurements available before diagnosis of diabetes, or the end of the study follow-up period, and with complete autoantibody level measurements for all three autoantibodies at seroconversion, were selected for our analysis (see electronic supplementary material [ESM] Fig. 1). This cohort (the ‘study cohort’) comprised 1403 children, of whom 523 (37.3%) developed diabetes (Table 1). All T1DI constituent studies were approved by the respective ethics review boards.

**Table 1:** Key characteristics of the study cohort

Variable	All (*n* = 1403)	Developed diabetes (*n* = 523)	Did not develop diabetes (*n* = 880)
Male	779 (55.5)	293 (56.0)	486 (55.2)
Age at seroconversion, initial visit (years)			
Mean ± SD	5.6 ± 4.2	3.6 ± 2.9	6.7 ± 4.3
Range	0.3–23.3	0.3–16.8	0.3–23.3
Age at seroconversion, confirmatory visit (years)			
Mean ± SD	6.1 ± 4.3	4.1 ± 3.1	7.2 ± 4.5
Range	0.5–23.9	0.5–18.7	0.5–23.9
Data source			
BABYDIAB	156 (11.1)	39 (7.5)	117 (13.3)
DAISY	178 (12.7)	64 (12.2)	114 (13.0)
DEW-IT	173 (12.3)	42 (8.0)	131 (14.9)
DIPIS	69 (4.9)	17 (3.3)	52 (5.9)
DIPP	827 (58.9)	361 (69.0)	466 (53.0)
HLA risk group			
A	333 (23.7)	183 (35.0)	150 (17.0)
B	666 (47.5)	248 (47.4)	418 (47.5)
C	182 (13.0)	46 (8.8)	136 (15.5)
D	219 (15.6)	46 (8.8)	173 (19.7)
Missing	3 (0.2)	0 (0.0)	3 (0.3)
Autoantibody-positive at seroconversion (initial visit)			
IAA	704 (50.2)	326 (62.3)	378 (43.0)
GADA	707 (50.4)	290 (55.4)	417 (47.4)
IA-2A	276 (19.7)	166 (31.7)	110 (12.5)
Autoantibody level at seroconversion (initial visit) (mULN)			
IAA	3.1 ± 8.2	4.5 ± 11.0	2.3 ± 6.7
GADA	5.7 ± 27.9	7.6 ± 34.2	4.6 ± 24.3
IA-2A	13.6 ± 59.8	25.2 ± 72.9	6.7 ± 52.0
Autoantibody-positive at seroconversion (confirmatory visit)			
IAA	787 (56.1)	364 (69.6)	423 (48.1)
GADA	865 (61.7)	381 (72.8)	484 (55.0)
IA-2A	416 (29.7)	278 (53.2)	138 (15.7)
Autoantibody level at seroconversion (confirmatory visit) (mULN)			
IAA	4.4 ± 12.6	7.4 ± 16.4	2.7 ± 9.2
GADA	9.5 ± 64.6	13.9 ± 75.7	6.9 ± 56.9
IA-2A	24.5 ± 75.7	50.4 ± 94.0	9.0 ± 57.0

Data are presented as *n* (%), means ± SD, or range

Percentages may not total to 100 because of rounding. Autoantibody-positive percentages may not total to 100 due to multiple positivity

### Laboratory measurements

The methods used by each study to measure IAA, GADA and IA-2A have been previously described [18]. Autoantibody levels for IAA, GADA, and IA-2A from the individual T1DI constituent studies were converted to multiples of the upper limit of normal (mULN) to facilitate comparisons, and were combined for analysis as previously described [17]. All mULN values, regardless of whether they were above or below the autoantibody positivity threshold, were included in the analysis. The autoantibody levels were natural log-transformed before use in the prediction models. Autoantibodies to zinc transporter 8 (ZnT8A) were not consistently measured across all constituent T1DI studies, and are therefore not included in our analysis.

For each IAb type, seroconversion was defined as the first appearance of positive autoantibody test results (for the same autoantibody type) in at least two consecutive samples, regardless of the time interval between the visits. The first and second of these two consecutive visits are referred to as the initial visit and the confirmatory visit, respectively (ESM Fig. 2). The time intervals, in years, between the initial and confirmatory visits for IAA, GADA and IA-2A were 0.4± 0.5, 0.5±0.5 and 0.4±0.7, respectively (mean±SD). The mean age of the participants, the percentage of participants positive for each autoantibody type, and mean autoantibody levels at the initial and confirmatory visits are shown in Table 1.

HLA genotypes from individual studies were harmonised into four risk groups: A, B, C and D (ordered by decreasing risk, e.g. A=DR4-DQ8/DR3-DQ2.5 represents the highest risk) as previously described [18].

### Outcome definition

Diagnosis of type 1 diabetes was based on the WHO and ADA criteria [24]. The main outcome of interest was the diagnosis of diabetes within a given follow-up period (*T* years) starting at a specified time point (‘time 0’) at the confirmatory visit (ESM Fig. 2) and *W* years after the confirmatory visit (ESM Fig. 3). Children diagnosed with diabetes before ‘time 0’ were excluded. Children diagnosed with diabetes after the given follow-up period were treated as not diagnosed with diabetes.

### Statistical analyses

All analyses used multivariate logistic regression prediction models with inverse probability censored weighting (IPCW) to account for the censored observations [25]. To make efficient use of the data and to obtain performance estimates from test data independent of the training data, 10-fold cross-validation was used [26]. This was done by randomly splitting the dataset into ten equally sized partitions, using nine of the partitions to train the prediction model and the remaining partition to test the model, and repeating this ten times using different 9:1 groupings of the partitions each time. The final performance was then computed by averaging the performance of the ten models. Discriminative power for disease, i.e. prediction performance, was estimated using the IPCW concordance index (C index) to adjust for censoring [27], and 95% CI were estimated via bootstrap [28]. The C index is a generalisation of the more commonly used area under the receiver operating curve (ROC-AUC) that can account for censored data; it measures the model’s ability to correctly provide a reliable ranking of the survival times based on the individual risk scores. ORs derived from the beta coefficients of the fitted logistic regression models were used to assess the strength of association between the covariates and the diabetes outcome. A *p* value <0.01 (two-sided Wald test) was considered statistically significant. The following logistic regression model assumptions were checked and confirmed on the most complex model considered (ESM Fig. 4): binary response variable, linearity in the logit for continuous predictor variables, lack of strongly influential outliers, absence of severe multicollinearity, independence of errors and adequate number of events per predictor variable. We believe that the assumptions would continue to hold for the simpler models fitted using subsets of the data.

Three analyses were performed as described below, each focused on addressing a specific question.

#### How well does IAb information at seroconversion predict future diabetes onset?

The prediction task for this analysis is illustrated in ESM Fig. 2. ‘Time 0’ is the time point when the prediction was made, i.e. the time of the confirmatory visit. Information obtained at or prior to ‘time 0’, such as baseline covariates, information from the initial visit and information from the confirmatory visit, were used as covariates in the prediction model. The outcome was determined based on the presence (1) or absence (0) of a diabetes diagnosis in the 10-year follow-up period.

To characterise and quantify the utility of IAb positivity indicators and IAb levels in predicting diabetes onset, a series of nine prediction models as defined below with different sets of covariates were evaluated and compared: (1) baseline covariates (i.e. data source, sex, diabetes family history, HLA risk group, age at initial visit, age at confirmatory visit); (2) IAb positivity indicators from the initial visit; (3) IAb positivity indicators from the confirmatory visit; (4) IAb positivity indicators from both initial and confirmatory visits; (5) IAb levels from the initial visit; (6) IAb levels from the confirmatory visit; (7) IAb levels from both initial and confirmatory visits; (8) baseline covariates plus the IAb positivity indicators from both visits; and (9) baseline covariates plus the IAb levels from both visits.

#### How does prediction performance change as the follow-up period varies?

To characterise how prediction performance changes as the follow-up period varies, we performed a series of analyses using the same prediction task illustrated in ESM Fig. 2 but varying the length of the follow-up period (*T*) from 1 to 15 years in 1-year increments. For each value of *T*, the cohort was updated (ESM Fig. 5) and used to train and evaluate two prediction models: one that used the baseline covariates plus the IAb levels from both initial and confirmatory visits, and another that used only the IAb levels from the confirmatory visit. Prediction performance (C index) as a function of the follow-up period (*T*) was then assessed and compared across the two models.

#### What is the predictive value of additional IAb information measured after confirmed seroconversion?

To quantify the predictive value of IAb information measured after confirmed seroconversion, we modified the prediction task as illustrated in ESM Fig. 3. A third visit, *W* years after the confirmatory visit, was added, and ‘time 0’ (the prediction start time) was moved to this later time point. We explored a range of nine values for *W*: 0.25, 0.5, 1.0, 1.5, 2.0, 2.5, 3.0, 4.0 and 5.0 years. The confirmatory visit corresponds to *W*=0. IAb information from the visit immediately prior to the specified third time point was used. The total number of diagnosed and not diagnosed participants for the various values of *W* are shown in ESM Fig. 6. The cohort was updated for each of the 15×9=135 pairs of values for follow-up period (*T*)×interval from confirmatory visit to the next test (*W*), and a prediction model using the GADA, IA-2A and IAA levels from ‘time 0’ as covariates was trained and evaluated. The prediction performance (C index) and ORs of the IAb covariates, as a function of *T* and *W*, were then assessed.

Analyses were performed using Python (scikit-learn, scikit-survival) and R software (survival, survminer, statsmodels) [29, 30].

## Results

### IAb levels add to IAb positivity when predicting diabetes onset from seroconversion

Figure 1 shows the diabetes prediction performance for models using the various covariate sets. An initial model using a 10-year follow-up period with baseline covariates had a C index of 0.607 (95% CI 0.584, 0.628). Significant improvement was observed after adding IAA, GADA, IA-2A positivity indicators from the initial and confirmatory visits: 0.722 (95% CI 0.707, 0.736). Adding the autoantibody levels instead resulted in even better performance: C index 0.756 (95% CI 0.744, 0.767) (Fig. 1a). Interestingly, the C index was 0.757 (95% CI 0.753, 0.760) for a model that considered only the IAb levels at the confirmatory visit and no baseline covariates (Fig. 1b). Overall, models using IAb information from the confirmatory visit performed significantly better than models using information from just the initial visit (*p*<0.001). Models using information from both the initial and confirmatory visits did not outperform models using information from just the confirmatory visit. Models using IAb levels performed significantly better than models using IAb positivity indicators (*p*<0.001). Adding baseline covariates to the IAb information, whether positivity indicators or levels, did not improve prediction performance.

ESM Fig. 4 shows a Forest plot of the multivariable logistic regression model for predicting type 1 diabetes onset using the ‘baseline+IAb levels (initial+confirmatory)’ covariate set. IAb levels from the confirmatory visit for all three IAb types were highly significant (*p*<0.0001) with ORs of 1.36 (95% CI 1.25, 1.47), 1.32 (95% CI 1.22, 1.44) and 1.15 (95% CI 1.07, 1.24) for IA-2A, GADA and IAA, respectively. GADA and IA-2A levels from the initial visit were not significant but IAA levels were significant (OR 1.08 [95% CI 1.02, 1.15]; *p*=0.009). The baseline covariate HLA group A (highest risk) was also a significant predictor, probably due to the heterogeneous population of single IAb-positive and multiple IAb-positive participants at seroconversion. The data source features DS_DAISY and DS_DIPIS were also statistically significant compared with the reference DS_DIPP.

### Prediction performance was better for shorter follow-up periods (i.e. rapid-onset diabetes) and remained reasonable for longer follow-up periods

**Fig. 2:**
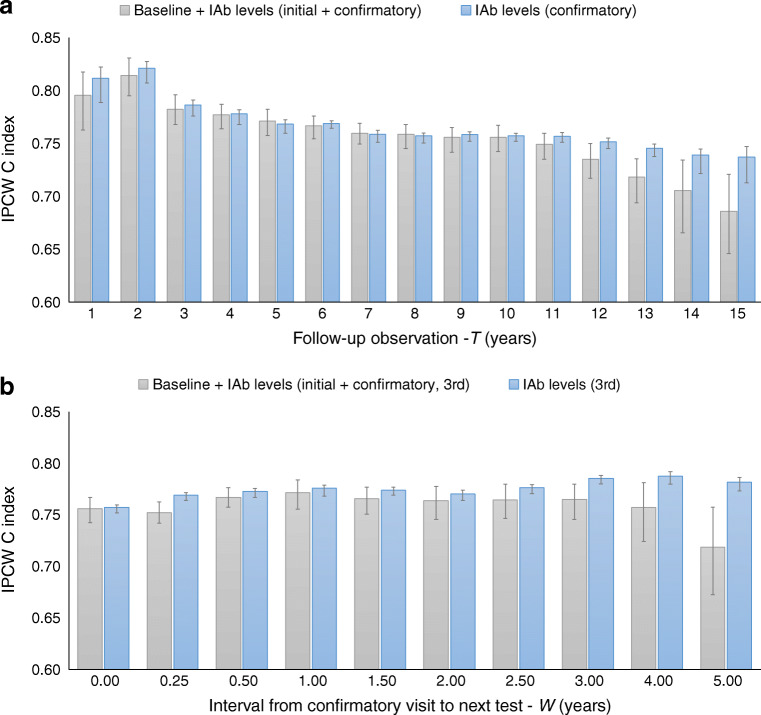
Comparison of type 1 diabetes prediction performance (IPCW concordance index [C index] with 95% CI) for two models. The first model (blue) used only the most recent IAb levels at the prediction start time (‘time 0’); the second model (grey) added baseline covariates and IAb levels from the initial and confirmatory visits to the most recent IAb levels. (**a**) Performance for various follow-up periods (*T*) ranging from 1 to 15 years. The prediction start time (‘time 0’) was the seroconversion confirmatory visit. (**b**) Performance for various test intervals (*W*) ranging from 0.25 to 5 years (*W*=0 is the confirmatory visit). In this analysis, the prediction time point (‘time 0’) was the time of the third visit (confirmatory visit+*W*). The follow-up period starts from the prediction time point and was fixed at 10 years

Figure 2a shows type 1 diabetes prediction performance for various follow-up periods (*T*) ranging from 1 to 15 years. The model that used only the IAb levels from the confirmatory visit (blue) had equivalent or better performance than the model that used the baseline covariates plus the IAb levels from both initial and confirmatory visits (grey). Prediction performance (C index) was 0.812 (95% CI 0.789, 0.822) at 1 year and 0.821 (95% CI 0.807, 0.827) at 2 years, and decreased slowly from 0.786 (95% CI 0.776, 0.791) at 3 years to 0.757 (95% CI 0.752, 0.760) at 11 years and finally to 0.737 (95% CI 0.713, 0.747) at 15 years. Although diabetes prediction performance decreased with longer follow-up periods, prediction performance was high for short follow-up periods (i.e. rapid-onset diabetes) and remained reasonable up until 11 years of follow-up.

### A third IAb test added to predictive power, and a suitable interval between confirmed seroconversion and the third test was approximately 1.5 years

Figure 2b shows the type 1 diabetes prediction performance for various intervals from the confirmatory visit to the next IAb test (*W*) ranging from 0.25 to 5 years (*W*=0 is the confirmatory visit). Again, the model that used only the IAb levels from the third visit (blue) consistently performed as well as or better than the model that used the baseline covariates plus the IAb levels from the initial, confirmatory and third visits (grey).

**Fig. 3:**
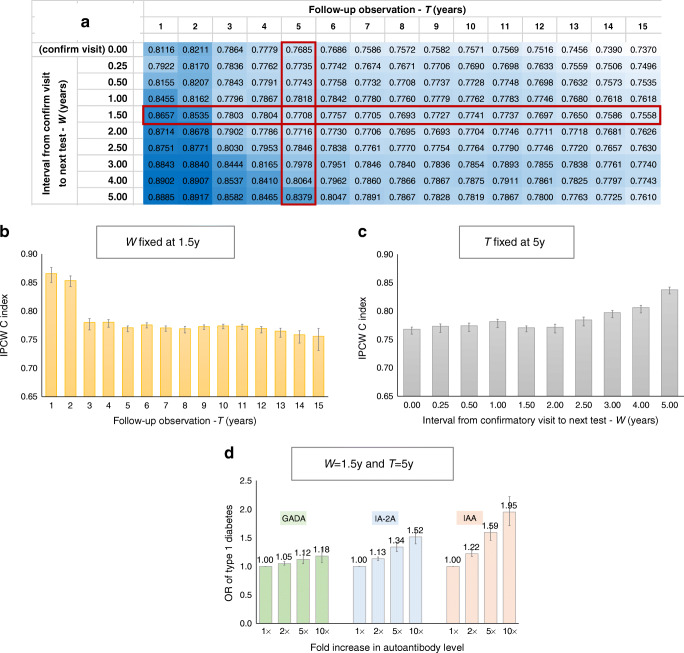
(**a**) Type 1 diabetes prediction performance (IPCW concordance index [C index]) for various follow-up periods (*T*), ranging from 1 to 15 years, along the horizontal axis, and various intervals from confirmatory visit to the next test (*W*), ranging from 0.25 to 5 years, along the vertical axis (*W*=0 is the confirmatory visit). In this analysis, the prediction time point (‘time 0’) was the time of the third visit (confirmatory visit+*W*). The follow-up period starts from the prediction time point. All prediction models used just three covariates: GADA, IA-2A and IAA levels from the third visit. Darker shading indicates better performance. A standalone version of the table can be found as ESM Table 1. (**b**) Type 1 diabetes prediction performance for various follow-up periods (*T*) ranging from 1 to 15 years, with the test interval *W* fixed at 1.5 years. (**c**) Type 1 diabetes prediction performance for various test intervals (*W*) ranging from 0.25 to 5 years (*W*=0 is the confirmatory visit), with follow-up period (*T*) fixed at 5 years. (**d**) ORs for developing type 1 diabetes for a 1-, 2-, 5- and 10-fold increases in the levels of GADA, IA-2A and IAA separately for a prediction time point of *W*=1.5 years and a follow-up period (*T*) of 5 years. Confirm, confirmatory; y, years

Figure 3a and ESM Table 1 show type 1 diabetes prediction performance as a function of both the duration of the follow-up period (*T*) and the interval from confirmatory visit to the next IAb test (*W*), with *T* ranging from 1 to 15 years and *W* ranging from 0.25 to 5 years. The prediction models used for this analysis only included three covariates: the GADA, IA-2A and IAA levels from the latest visit (i.e. the ‘time 0’ prediction time point in ESM Fig. 3).

For a fixed value of *W* (i.e. across each row), the prediction performance decreased as the follow-up period *T* increased. An example is shown for *W*=1.5 years in Fig. 3b. For a given value of *T* (i.e. along each column), the prediction performance improved as the test interval *W* increased. An example is shown for *T*=5 years in Fig. 3c. For short follow-up periods (i.e. *T*≤5 years), performance continued to improve with increasing *W*. However, for longer follow-up periods (i.e. *T* >5 years), performance improved as *W* increased to 1.0–1.5 years and plateaued thereafter. Given this, a reasonable trade-off between practical testing intervals and improved prediction accuracy is *W*=1.5 years.

To understand better how the strength of association of the IAb levels and diabetes outcome varied with the duration of the follow-up period (*T*) and the test interval (*W*), ESM Figs 7 and 8 show the ORs and corresponding beta coefficients, respectively, for the GADA, IA-2A and IAA autoantibody levels measured at ‘time 0’ as a function of *T* and *W*. When the follow-up period was short (*T*≤5 years), GADA levels showed a low but steady association with diabetes (OR 1.0–1.1) for all combinations of *W* and *T*; IA-2A levels showed a range of associations from none to moderate (OR 1.0–1.3) that increased with larger values of *T* and decreased with larger values of *W*; IAA levels had moderate to strong association with diabetes (OR 1.2–1.7) that increased with larger values of *T* and larger values of *W*. When the follow-up period was long (*T*>5 years), GADA levels had a low to moderate association with diabetes (OR 1.1–1.3) that increased with larger values of *T* and decreased with larger values of *W*; IA-2A levels showed a moderate association with diabetes (OR 1.2–1.4) that increased with larger values of *T* and decreased with larger values of *W*; IAA levels demonstrated a moderate to strong association with diabetes (OR 1.3–1.6) that was reasonably steady with larger values of *T* and increased with larger values of *W*.

Another way to look at the strength of association of the IAb levels and diabetes outcome is to estimate the ORs of developing type 1 diabetes within a given follow-up period (*T*) for a specific *n*-fold increase in the level of each autoantibody type (ESM Section 1). Figure 3d shows the ORs for the development of type 1 diabetes for 1-, 2-, 5- and 10-fold increases in GADA, IA-2A and IAA levels, with a test interval of *W*=1.5 years and a follow-up period of *T*=5 years. An increase in GADA levels resulted in a small increase in the OR (e.g. 12% for a 5-fold increase). For IA-2A, an increase in levels resulted in a moderate increase in the OR (e.g. 34% for a 5-fold increase). An even stronger effect was observed for IAA (e.g. a 59% increase in OR for a 5-fold increase).

## Discussion

This study demonstrated that IAb information at the time of seroconversion and thereafter may be used for robust predictions of both rapid-onset type 1diabetes and slow type 1 diabetes progression in autoantibody-positive children. Furthermore, the study also revealed that the distinct types of IAb and the number and timing of their measurements affected the prediction model in different ways.

IAb information at the time of confirmation of a newly developed autoantibody response (i.e. the confirmatory visit) performed better than IAb information obtained at the very first detection of this autoantibody (i.e. the initial visit) and about the same as using information from both visits, suggesting that the later (and more ‘mature’) autoantibody response may be more robust, and captures the most salient information for diabetes onset prediction. In general, using IAb levels improved prediction over just using IAb positivity indicators, consistent with observations from previous studies [16, 31]. Furthermore, adding the baseline covariates to the IAb information did not improve prediction performance, suggesting that the nature of these covariates was inferior in predicting diabetes risk compared with IAb information. However, other studies have shown that using more detailed genetic information, and other IAb characteristics such as epitope and affinity, which are complementary to IAb type and level, improves prediction performance [32]. We believe that including such complementary information in our model would also help improve performance. However, in order to obtain this kind of information, additional testing and analyses would have to be performed.

By varying the length of the follow-up period (*T*), we gained insight into how well rapid and slow progression to diabetes can be predicted using IAb levels. Prediction performance was significantly better for short follow-up periods (i.e. rapid progression to diabetes) than for longer follow-up periods (i.e. overall diabetes progression, including both rapid and slow progression) but prediction became more challenging as the follow-up period increased. Similar patterns of decreasing prediction performance with longer prediction windows have been observed for other clinical outcomes such as hypoglycaemia [33], heart failure [34] and mortality [35]. However, prediction performance remained high (i.e. C index >0.75) for follow-up periods up to 11 years, suggesting that IAb levels around the time of seroconversion are robust predictors of progression to diabetes within the subsequent decade.

By changing the prediction time point (‘time 0’) to after the seroconversion confirmatory visit, and varying the time interval (*W*) for this visit, we were able to assess the behaviour and characteristics of the various IAb types and the timing of IAb measurements required for robust prediction of diabetes onset. We explored various combinations of covariates, and found that a child’s baseline characteristics and previous IAb information were not critical for diabetes risk prediction if current IAb information was available. This suggests that using only the most recent IAb levels may be sufficient for diabetes onset prediction, which, in addition to simplifying the model, is also closer to actual clinical situations where an individual’s seroconversion time may not be precisely known and the IAb levels at seroconversion may not be available.

For longer follow-up periods (*T*>5 years), using IAb levels from a visit 1.0–1.5 years after the confirmatory visit improved prediction performance, but using IAb levels beyond that time frame did not improve the prediction performance further. This suggests that there are important changes in the IAb levels within approximately 1.5 years after seroconversion that are useful for predicting diabetes onset. This aligns with a recommendation from a prior study that diabetes risk stratification based on IAb levels should focus on time points soon after seroconversion [9]. However, for shorter follow-up periods (*T* ≤5 years), prediction performance continued to improve with larger values of *W*, indicating that the latest IAb levels remained important for predicting rapid-onset diabetes risk.

For both IAA and IA-2A with longer follow-up periods (*T* >5 years), the most recent IAb levels measured after seroconversion remained moderate to strong predictors of diabetes, regardless of how long after seroconversion they were measured. IAA levels have shown a consistent association with diabetes progression, whether the level was measured at the time of seroconversion in birth cohort studies [7, 12, 13] or in cross-sectional studies [6]. Similarly, a positive association between higher IA-2A levels and progression to diabetes has also been repeatedly observed, both in birth cohorts [13] and cross-sectional studies with older participants [6]. However, GADA levels more than 1 year after seroconversion were not as useful for predicting diabetes onset as levels measured shortly after the time of seroconversion. Results from cross-sectional studies with older autoantibody-positive participants did not find a significant association between GADA levels and diabetes [6]. In the TEDDY study, it was observed that GADA levels had a positive association with disease only in the first 12 months after seroconversion [9].

With shorter follow-up periods (*T*≤5 years), IA-2A levels closer to the time of seroconversion appear to be better predictors of rapid-onset diabetes risk than levels measured later. In contrast, recently measured IAA levels appear to be stronger predictors of rapid-onset diabetes risk than ones measured around the time of seroconversion. GADA levels had a low association with diabetes, regardless of when they were measured. For example, when *W*=1.5 years and *T*=5 years, the changes in the OR for developing type 1 diabetes resulting from a 5-fold increase in the level of GADA, IA-2A and IAA were 12%, 34% and 59%, respectively (Fig. 3d). Because all values for IAb levels were included in the analysis regardless of positivity status, and many of the IAb measurements were below the positivity threshold at the respective prediction time point (ESM Fig. 7), these effects should be considered conservative estimates.

Although we found that GADA levels had a low to moderate association with diabetes, the strength of the association increased with longer follow-up periods (i.e. were more useful in predicting slower progression to diabetes). This is consistent with other studies that found that GADA, as a primary autoantibody, is associated with a slower progression to diabetes compared with other autoantibodies [36, 37]. In contrast, IAA and IA-2A levels showed a moderate to strong association with diabetes, with IAA contributing particularly to the prediction of rapid progression to diabetes. This is consistent with several previous findings: one study found that infants in whom IAA was the first autoantibody detected were more likely to develop diabetes within the first 2 years than infants with other autoantibodies at seroconversion [36], another study found that lower initial IAA levels independently predicted slower progression to diabetes [10], and another study showed that IA-2A levels have a stable and consistent association with risk of progression to diabetes after seroconversion [9].

This study has some limitations. First, the autoantibody levels were measured using different assays across the study sites. Although the levels were harmonised, some residual biases may remain. In addition, the current data are based on radiobinding assay results as newer assay technologies [38–40] were not available when the data were collected. Second, due to differences in the visit intervals in the study protocols, it is possible that the actual time of the earliest IAb positivity was missed, with the consequence that the measurement time is biased. Off-schedule visits may also affect the timing of the initial and confirmatory visits. Third, predominantly white children with increased genetic and familial risk for development of islet autoimmunity and diabetes were enrolled into the studies, which may limit the generalisability of the results to the general population. Fourth, the results have not been validated using external independent datasets.

There are several possible directions for future work. First, the analyses should be replicated using higher time-resolution datasets with more frequent prospective follow-up (e.g. TEDDY [41]). Second, validation in independent cohorts with broader inclusion criteria (e.g. the Fr1da [42] or ASK [43] studies) should be undertaken. Third, the utility of IAb levels as a continuous variable should be explored in other tasks such as modelling of diabetes disease progression [44, 45].

In summary, this study used harmonised IAb levels across multiple birth cohorts and quantified their utility for predicting type 1 diabetes onset in IAb-positive children. We found that IAb levels add to IAb positivity when predicting diabetes onset from seroconversion, that predictive power was maintained when using IAb levels alone, that prediction performance was better for shorter follow-up periods (i.e. rapid-onset diabetes) but remained reasonable for longer follow-up periods (up to 11 years), and that a third IAb test added to predictive power and that a suitable interval between confirmed seroconversion and the third test was approximately 1.5 years. Our findings suggest the utility of monitoring IAb levels approximately 1.5 years after seroconversion, especially IAA and IA-2A, as they appear to be important predictors of diabetes risk in children. The results of this study may contribute to improved risk counselling for families of affected children and improved screening for participants for intervention therapy trials aimed at preventing or delaying progression to clinical diabetes.

## Supplementary information

ESM (PDF 825 kb)

## Data Availability

The data that support the findings of this study are available from each of the five study groups (DiPiS, BABYDIAB, DIPP, DEW-IT and DAISY) but restrictions apply to the availability of these data, which were used under licence for the current study, and so are not publicly available. However, data are available from the authors upon reasonable request and with permission from the five study groups.

## Abbreviations

GADA: Glutamic acid decarboxylase autoantibodies
IA-2A: Insulinoma-associated antigen-2 autoantibodies
IAA: Insulin autoantibodies
IAb: Islet autoantibody
IPCW: Inverse probability censored weighting
mULN: Multiples of upper limit of normal
T1DI: Type 1 Diabetes Intelligence
