# Supplementary material for: Quantifying the utility of islet autoantibody levels in the prediction of type 1 diabetes in children
Source: Diabetologia. 2022 Oct 5;66(1):93–104. doi: 10.1007/s00125-022-05799-y (PMC9729160; doi:10.1007/s00125-022-05799-y)
Supplement: Supplementary file 1 — (PDF 825 kb) [file 125_2022_5799_MOESM1_ESM.pdf]

Supplementary Appendix

Supplement to Ng et al., Quantifying the utility of islet autoantibody levels in the prediction of type 1 diabetes in children

ESM Section 1: Estimating the odds ratio of developing type 1 diabetes for an N-fold change in the level of each autoantibody type 2
ESM Section 2: The T1DI Study Group 3
ESM Figure 1 4
ESM Figure 2 5
ESM Figure 3 6
ESM Figure 4 7
ESM Figure 5 8
ESM Figure 6 9
ESM Figure 7 10
ESM Figure 8 11
ESM Table 1 12

ESM Section 1: Estimating the odds ratio of developing type 1 diabetes for an N-fold change in the level of each autoantibody type

Since we fit a logistic regression model to the natural log transformed harmonized autoantibody levels (*x*) to predict the development of type 1 diabetes, the log odds can be expressed as:

$$\mathrm{t} = \log\left(\frac{\mathrm{p}}{1-\mathrm{p}}\right) = \beta_0 + \beta_{\mathrm{GADA}} * \ln(\mathrm{x}_{\mathrm{GADA}}) + \beta_{\mathrm{IAA}} * \ln(\mathrm{x}_{\mathrm{IAA}}) + \beta_{\mathrm{IA-2A}} * \ln(\mathrm{x}_{\mathrm{IA-2A}})$$

where β_0, β_{GADA}, β_{IAA}, and β_{IA-2A} are the beta coefficients of the fitted logistic regression model, x_{GADA}, x_{IAA}, and x_{IA-2A} are the harmonized autoantibody levels for GADA, IAA, and IA-2A, respectively. The odds of developing type 1 diabetes can then be written as $\mathrm{e}^{t} = \frac{p}{1-p}$:

$$\begin{aligned} &= \exp(\beta_0) \cdot \exp\left(\beta_{GADA} \cdot ln(x_{GADA})\right) \cdot \exp(\beta_{IAA} \cdot \ln(x_{IAA})) \cdot \exp(Beta_{IA-2A} \cdot \ln(x_{IA-2A})) \\ &= \exp(\beta_0) \cdot \exp(\ln(x_{GADA}))^{\beta_{GADA}} \cdot \exp(\ln(x_{IAA}))^{\beta_{IAA}} \cdot \exp(\ln(x_{IA-2A}))^{\beta_{IA-2A}} \\ &= \exp(Beta_0) \cdot (x_{GADA})^{\beta_{GADA}} \cdot (x_{IAA})^{\beta_{IAA}} \cdot (x_{IA-2A})^{\beta_{IA-2A}} \end{aligned}$$

If we hold two of the autoantibody levels fixed (e.g., x_{IAA} and x_{IA-2A}), the odds for developing type 1 diabetes for two different values of GADA (x_{GADA} and y_{GADA}) are:

$$\mathrm{odds}(\mathrm{x}_{\mathrm{GADA}}) = exp(\beta_0) \cdot (x_{GADA})^{\beta_{GADA}} \cdot (x_{IAA})^{\beta_{IAA}} \cdot (x_{IA-2A})^{\beta_{IA-2A}}$$

$$\mathrm{odds}(y_{GADA}) = exp(\beta_0) \cdot (y_{GADA})^{\beta_{GADA}} \cdot (x_{IAA})^{\beta_{IAA}} \cdot (x_{IA-2A})^{\beta_{IA-2A}}$$

and the odds ratio of developing type 1 diabetes for the two different GADA levels is:

$$\frac{\mathrm{odds}(\mathrm{y}_{\mathrm{GADA}})}{\mathrm{odds}(\mathrm{x}_{\mathrm{GADA}})} = \frac{(\mathrm{y}_{\mathrm{GADA}})^{\beta_{\mathrm{GADA}}}}{(\mathrm{x}_{\mathrm{GADA}})^{\beta_{\mathrm{GADA}}}} = \left(\frac{\mathrm{y}_{\mathrm{GADA}}}{\mathrm{x}_{\mathrm{GADA}}}\right)^{\beta_{\mathrm{GADA}}}$$

Similarly for IAA and IA2A, we have these expressions for the odds ratios:

$$\frac{odds(y_{IAA})}{odds(x_{IAA})} = \frac{(y_{IAA})^{\beta_{IAA}}}{(x_{IAA})^{\beta_{IAA}}} = \left(\frac{y_{IAA}}{x_{IAA}}\right)^{\beta_{IAA}}$$

$$\frac{odds(y_{IA-2A})}{odds(x_{IA-2A})} = \frac{(y_{IA-2A})^{\beta_{IA-2A}}}{(x_{IA-2A})^{\beta_{IA-2A}}} = \left(\frac{y_{IA-2A}}{x_{IA-2A}}\right)^{\beta_{IA-2A}}$$

Supplemental Figure S10 plots the odds ratios (ORs) of developing type 1 diabetes for an N = 1-, 2-, 5-, and 10-fold increase in the level of GADA, IAA, and IA-2A separately for an observation interval of W=1 year and a follow-up time of T=5 years. In other words, $y_{GADA} = \mathrm{N} \cdot x_{GADA}$, $y_{IAA} = \mathrm{N} \cdot x_{IAA}$, and $y_{IA-2A} = \mathrm{N} \cdot x_{IA-2A}$ for N=1, 2, 5, and 10. The beta coefficient values are shown in Supplemental Figure S9.

ESM Section 2: The T1DI Study Group

BABYDIAB: Anette G. Ziegler, M.D., Ezio Bonifacio Ph.D., Peter Achenbach, M.D., Christiane Winkler, Ph.D.; Forschergruppe Diabetes e.V. and Institute of Diabetes Research, Helmholtz Zentrum München, German Research Center for Environmental Health, Munich-Neuherberg, Germany der TU München, Munich, Germany

DAISY: Marian Rewers, M.D., Ph.D., Brigitte I. Frohnert, M.D., Ph.D., Jill Norris, Ph.D., Andrea Steck, M.D., Kathleen Waugh, M.P.H., Liping Yu, M.D.; University of Colorado, Anschutz Medical Campus, Barbara Davis Center for Diabetes.

DEW-IT: William A. Hagopian, M.D., Ph.D., Michael Killian, Rachel Hervey; Pacific Northwest Research Institute.

DiPiS: Åke Lernmark, Ph.D., Helena Elding Larsson, M.D., Ph.D., Markus Lundgren, M.D., Ph.D., Marlena Maziarz, Ph.D., Lampros Spiliopoulos, Josefin Jönsson; Department of Clinical Sciences Malmö, Lund University.

DIPP: [1]Riitta Veijola, M.D., Ph.D., [2]Jorma Toppari, M.D., Ph.D., [2]Jorma Ilonen, M.D., Ph.D., [3,4]Mikael Knip, M.D., Ph.D., [3,5]Heikki Hyöty, M.D., Ph.D; [1]University of Oulu and Oulu University Hospital,[2]University of Turku and Turku University Hospital, [3]Tampere University Hospital, [4]University of Helsinki, [5]Hospital District of Pirkanmaa.

IBM: Vibha Anand, Ph.D., Mohamed Ghalwash, Ph.D., Bin Liu, Ph.D., Kenney Ng, Ph.D., Zhiguo Li, Ph.D., Ying Li, Ph.D., B.C. Kwon, Ph.D., Harry Stravropoulos, M.S., Eileen Koski, M.Phil, Ashwani Malhotra, Ph.D., Shelley Moore, Jianying Hu, Ph.D.

JDRF: Jessica Dunne, Ph.D., Olivia Lou, Ph.D, Frank Martin, Ph.D.

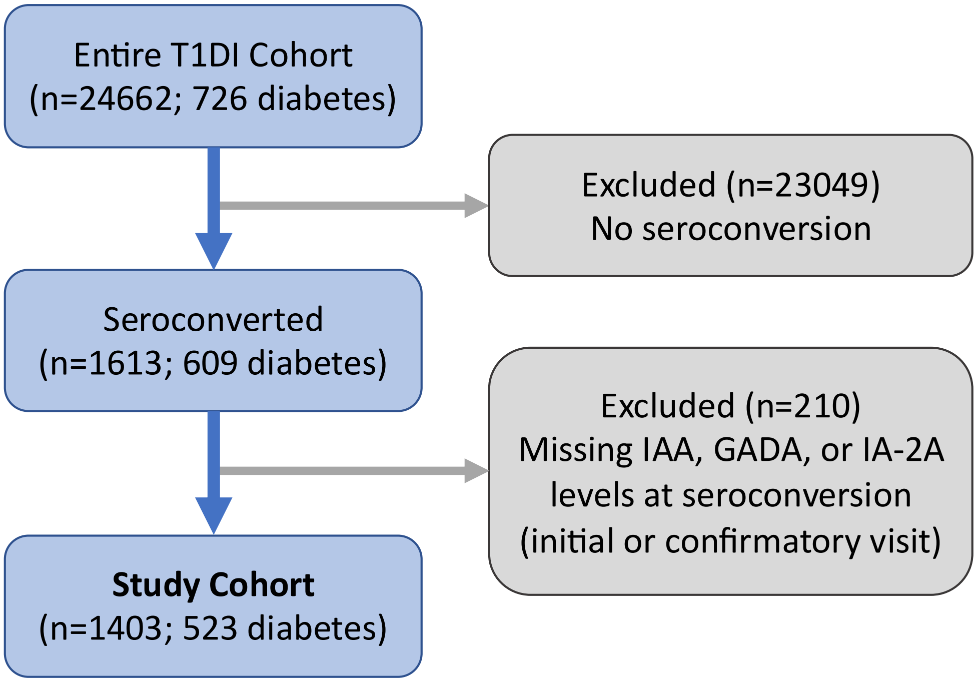

ESM Figure 1: Study cohort selection flowchart. IAA indicates insulin autoantibodies; GADA, glutamic acid decarboxylase autoantibodies; and IA-2A, insulinoma-associated antigen-2 autoantibodies.

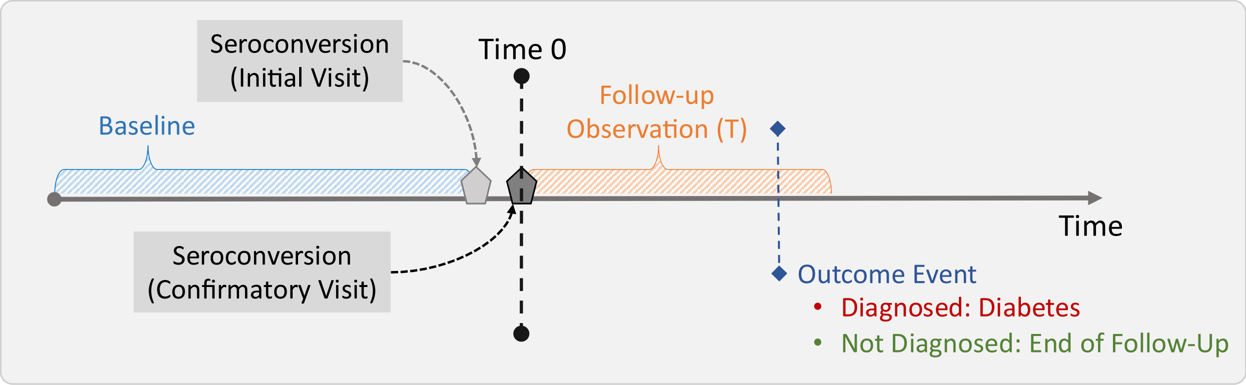

ESM Figure 2: Illustration of the relative temporal relationships between baseline, the initial visit of seroconversion, the confirmatory visit of seroconversion ("Time 0"), the follow-up observation (T), and the outcome event: either diabetes diagnosis or end of follow up.

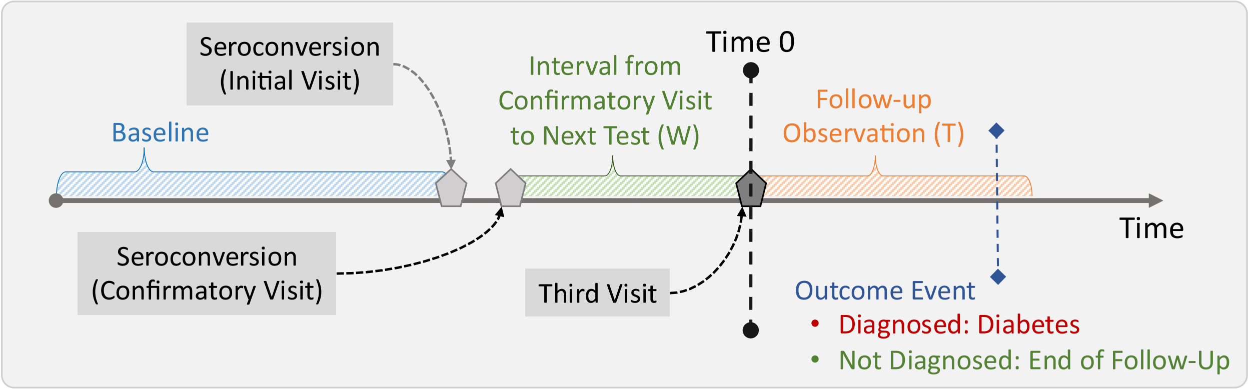

ESM Figure 3: Illustration of the relative temporal relationships between baseline, the initial visit of seroconversion, the confirmatory visit of seroconversion, a third visit with a time interval W years after the confirmatory visit ("Time 0"), the follow-up observation (T), and the outcome event: either diabetes diagnosis or end of follow up.

Variable	Coefficient	Odds Ratio (95% CI)	P-Value
DS_BABYDIAB	0.3871	1.47 (0.60-3.59)	0.395
DS_DAISY	0.8141	2.26 (1.20-4.24)	0.011
DS_DEWIT	0.0856	1.09 (0.63-1.88)	0.759
DS_DIPIS	-0.9019	0.41 (0.19-0.89)	0.024
DS_DIPP (reference)			
HLA_GROUP_A	1.1989	3.32 (2.28-4.83)	<0.0001
HLA_GROUP_B	0.2535	1.29 (0.96-1.72)	0.087
HLA_GROUP_C	0.4355	1.55 (0.96-2.50)	0.076
HLA_GROUP_D (reference)			
SEX_Female	0.0132	1.01 (0.76-1.34)	0.927
T1DFamilyHistory_Yes	0.1508	1.16 (0.61-2.22)	0.647
AgeSeroconversion_initial	0.0199	1.02 (0.78-1.34)	0.886
AgeSeroconversion_confirmatory	-0.2080	0.81 (0.62-1.06)	0.124
GADA_Level_initial	-0.0758	0.93 (0.86-1.00)	0.050
IA2A_Level_initial	0.0267	1.03 (0.94-1.13)	0.578
IAA_Level_initial	0.0813	1.08 (1.02-1.15)	0.009
GADA_Level_confirmatory	0.2804	1.32 (1.22-1.44)	<0.0001
IA2A_Level_confirmatory	0.3046	1.36 (1.25-1.47)	<0.0001
IAA_Level_confirmatory	0.1377	1.15 (1.07-1.24)	<0.0001

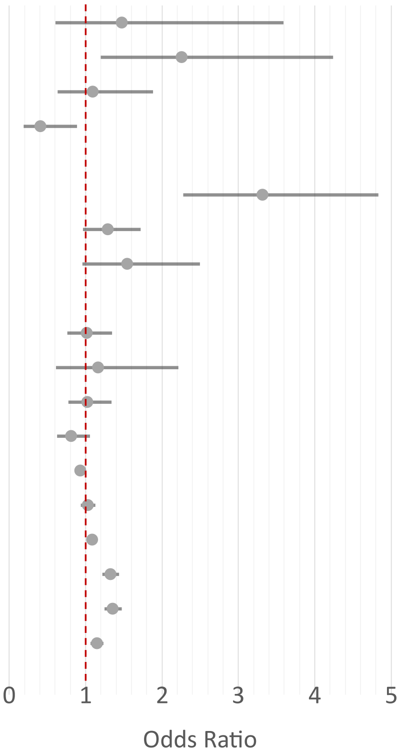

ESM Figure 4: Forest plot of the multivariable logistic regression model for predicting type 1 diabetes onset using the “Baseline + IAb Levels (initial + confirmatory)” covariate set. The prediction start time (“Time 0”) was the seroconversion confirmatory visit. Follow-up observation was T=10 years. The variable name, beta coefficients, odds ratios, 95% confidence intervals, and P-values, are shown. DS indicates data source.

The following set of logistic regression model assumptions were checked for this model:

- The dependent (response) variable (type 1 diabetes onset) was binary and only had two values: 0 and 1.
- Linearity of the independent (predictor) variables and log odds was checked using the Box-Tidwell test (p-values of the interaction terms were not significant and ranged from 0.054-0.925).
- Using a threshold of 0.005 for Cook's Distance, only 1.4% of the data points were highly influential outliers.
- There was no severe multicollinearity among the independent (predictor) variables as measured using the variance inflation factor (VIF ranged from 1.1-3.6 and were under the suggested threshold of 5.0).
- The observations were independent since they were from different subjects; a deviance residual versus index number plot also confirmed independence of the errors.
- The data set has 1403 samples (503 cases) which exceeded the guidance of a minimum of 10 samples (of the minority class) per predictor variable: (503 > 19*10).

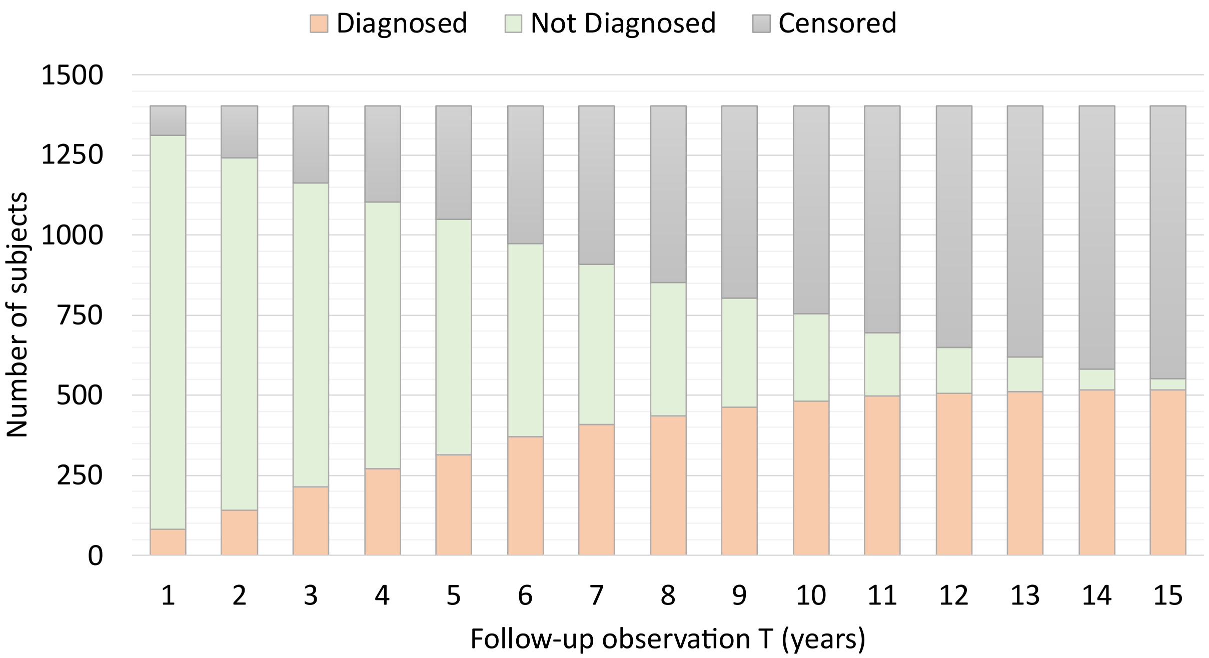

ESM Figure 5: Number of subjects diagnosed with diabetes, not diagnosed with diabetes, and censored (loss to follow-up) for different follow-up observations (T) ranging from T=1 year to T=15 years. The prediction start time ("Time 0") was the confirmatory visit of seroconversion.

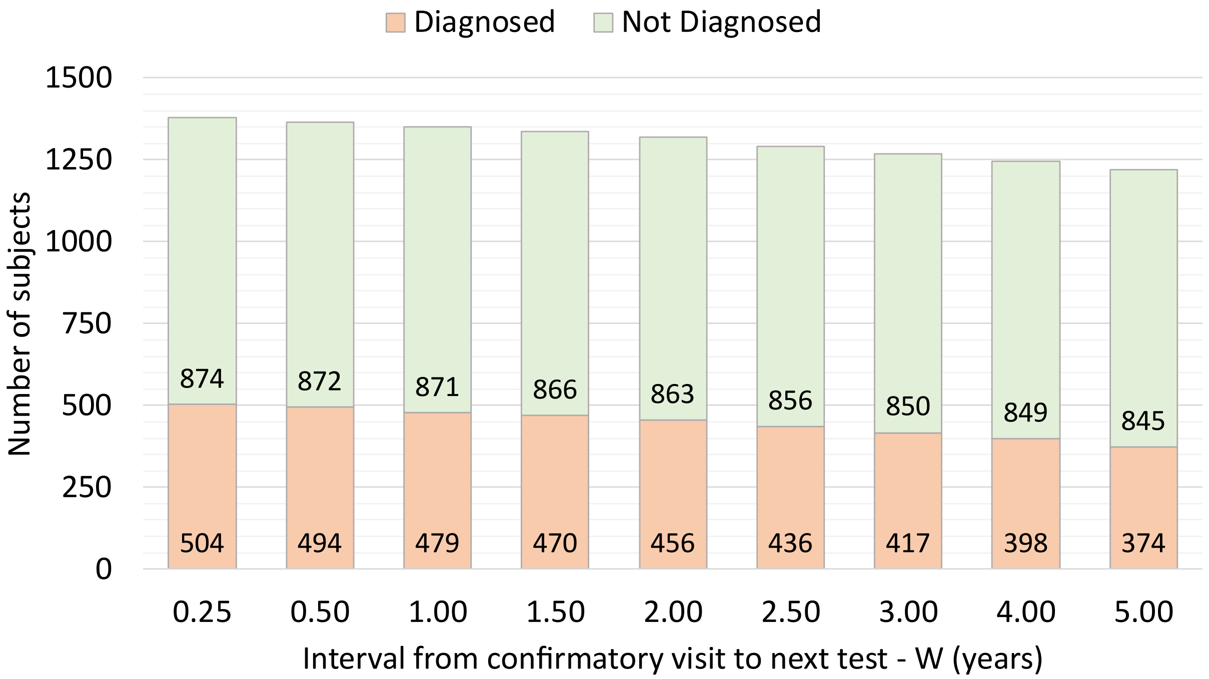

ESM Figure 6: Number of subjects diagnosed and not diagnosed with diabetes for different intervals from confirmatory visit to next test (W) ranging from 0.25 to 5 years based on the maximum follow-up observation available for each subject.

	IAb positivity	Interval from confirm visit to next test - W (years)	Follow-up observation - T (years) 1	2	3	4	5	6	7	8	9	10	11	12	13	14	15
GADA	61.65%	(confirm visit) 0.00	1.053	1.084	1.104	1.140	1.164	1.191	1.210	1.219	1.241	1.242	1.242	1.246	1.253	1.258	1.256
GADA	60.23%	0.25	1.048	1.080	1.096	1.128	1.150	1.180	1.200	1.218	1.239	1.238	1.239	1.241	1.249	1.258	1.267
GADA	57.76%	0.50	1.048	1.060	1.074	1.095	1.117	1.130	1.141	1.168	1.187	1.198	1.208	1.215	1.230	1.231	1.227
GADA	54.44%	1.00	1.069	1.077	1.070	1.090	1.091	1.103	1.111	1.142	1.160	1.168	1.170	1.177	1.186	1.196	1.195
GADA	52.92%	1.50	1.083	1.079	1.068	1.071	1.075	1.073	1.083	1.108	1.118	1.122	1.120	1.129	1.135	1.140	1.149
GADA	51.71%	2.00	1.095	1.095	1.073	1.073	1.070	1.076	1.078	1.104	1.113	1.118	1.114	1.126	1.129	1.143	1.145
GADA	50.54%	2.50	1.113	1.105	1.085	1.081	1.070	1.063	1.063	1.074	1.084	1.093	1.095	1.106	1.106	1.118	1.121
GADA	49.72%	3.00	1.111	1.106	1.099	1.086	1.074	1.073	1.076	1.088	1.102	1.106	1.100	1.112	1.122	1.130	1.128
GADA	48.60%	4.00	1.123	1.115	1.099	1.088	1.072	1.063	1.056	1.068	1.071	1.077	1.070	1.079	1.077	1.089	1.093
GADA	47.99%	5.00	1.133	1.137	1.111	1.098	1.092	1.077	1.079	1.079	1.090	1.084	1.081	1.082	1.080	1.090	1.098
IA-2A	29.65%	(confirm visit) 0.00	1.243	1.282	1.309	1.329	1.333	1.364	1.381	1.369	1.378	1.381	1.387	1.375	1.359	1.350	1.356
IA-2A	30.70%	0.25	1.179	1.223	1.260	1.288	1.304	1.358	1.370	1.364	1.389	1.390	1.412	1.398	1.379	1.372	1.375
IA-2A	31.99%	0.50	1.109	1.167	1.213	1.254	1.288	1.342	1.378	1.365	1.393	1.402	1.404	1.402	1.393	1.383	1.385
IA-2A	34.22%	1.00	*1.033*	1.098	1.152	1.199	1.243	1.298	1.346	1.344	1.369	1.379	1.400	1.401	1.383	1.380	1.383
IA-2A	35.78%	1.50	*0.999*	1.040	1.100	1.156	1.198	1.259	1.301	1.313	1.342	1.361	1.381	1.379	1.372	1.370	1.364
IA-2A	36.85%	2.00	*0.994*	*1.018*	1.076	1.140	1.185	1.246	1.294	1.313	1.342	1.371	1.383	1.382	1.383	1.382	1.383
IA-2A	36.69%	2.50	*0.980*	*1.007*	1.048	1.108	1.153	1.229	1.280	1.307	1.330	1.352	1.369	1.371	1.368	1.371	1.371
IA-2A	36.07%	3.00	*0.991*	*1.022*	1.048	1.112	1.165	1.234	1.283	1.312	1.341	1.355	1.385	1.391	1.390	1.386	1.386
IA-2A	37.45%	4.00	*0.976*	*1.010*	1.039	1.090	1.149	1.225	1.270	1.301	1.336	1.360	1.383	1.399	1.396	1.391	1.388
IA-2A	36.51%	5.00	*0.980*	*1.007*	1.045	1.094	1.114	1.191	1.239	1.265	1.301	1.327	1.354	1.360	1.366	1.360	1.361
IAA	56.09%	(confirm visit) 0.00	1.194	1.247	1.257	1.286	1.284	1.308	1.297	1.319	1.341	1.325	1.341	1.338	1.343	1.331	1.334
IAA	54.28%	0.25	1.186	1.234	1.247	1.271	1.291	1.305	1.304	1.334	1.362	1.347	1.357	1.355	1.359	1.360	1.358
IAA	49.63%	0.50	1.209	1.241	1.237	1.254	1.268	1.271	1.271	1.292	1.309	1.308	1.326	1.317	1.331	1.316	1.315
IAA	43.41%	1.00	1.306	1.323	1.305	1.336	1.325	1.314	1.308	1.305	1.303	1.291	1.291	1.292	1.285	1.274	1.280
IAA	41.09%	1.50	1.370	1.411	1.353	1.358	1.336	1.346	1.320	1.327	1.343	1.323	1.328	1.317	1.315	1.316	1.309
IAA	38.97%	2.00	1.402	1.445	1.429	1.395	1.365	1.349	1.328	1.324	1.319	1.323	1.333	1.330	1.346	1.340	1.339
IAA	37.07%	2.50	1.458	1.543	1.506	1.509	1.481	1.456	1.403	1.404	1.394	1.381	1.382	1.376	1.377	1.365	1.368
IAA	36.54%	3.00	1.500	1.583	1.588	1.560	1.499	1.472	1.424	1.410	1.411	1.401	1.422	1.417	1.416	1.402	1.402
IAA	33.36%	4.00	1.596	1.698	1.706	1.694	1.573	1.504	1.458	1.445	1.429	1.421	1.448	1.439	1.440	1.422	1.438
IAA	32.65%	5.00	1.583	1.710	1.727	1.728	1.736	1.572	1.495	1.483	1.437	1.416	1.424	1.415	1.442	1.430	1.422

ESM Figure 7: Odds ratios (ORs) of type 1 diabetes onset for the GADA, IA-2A, and IAA islet autoantibody levels at the third visit as a function of follow-up observation (T, ranging from 1 to 15 years along the horizontal axis) and interval from confirmatory visit to next test (W, ranging from 0.25 to 5 years along the vertical axis). The prediction time point ("Time 0") was the time of the third visit (confirmatory visit + W). The follow-up observation starts from the prediction time point. All prediction models used just 3 covariates: GADA, IA-2A, and IAA levels from the third visit. Statistically significant (p-value < 0.01) ORs are shown in black text; values in gray italics text are not statistically significant. The percentage of subjects positive for each islet autoantibody at "Time 0" is shown under the "IAb Positivity" column.

	IAb positivity	Interval from confirm visit to next test - W (years)	Follow-up observation - T (years) 1	2	3	4	5	6	7	8	9	10	11	12	13	14	15
GADA	61.65%	(confirm visit) 0.00	0.051	0.080	0.099	0.131	0.152	0.174	0.191	0.198	0.216	0.217	0.217	0.220	0.226	0.229	0.228
	60.23%	0.25	0.047	0.077	0.092	0.121	0.140	0.166	0.182	0.197	0.214	0.214	0.214	0.216	0.223	0.230	0.237
	57.76%	0.50	0.047	0.059	0.071	0.091	0.111	0.122	0.132	0.155	0.172	0.181	0.189	0.195	0.207	0.208	0.205
	54.44%	1.00	0.066	0.074	0.068	0.086	0.087	0.098	0.105	0.133	0.149	0.155	0.157	0.163	0.170	0.179	0.178
	52.92%	1.50	0.080	0.076	0.066	0.068	0.072	0.070	0.080	0.103	0.111	0.115	0.113	0.121	0.127	0.131	0.139
	51.71%	2.00	0.090	0.091	0.071	0.070	0.068	0.074	0.076	0.099	0.107	0.111	0.108	0.119	0.122	0.133	0.135
	50.54%	2.50	0.107	0.099	0.082	0.078	0.068	0.061	0.061	0.071	0.080	0.089	0.091	0.101	0.101	0.111	0.114
	49.72%	3.00	0.105	0.101	0.095	0.083	0.071	0.070	0.073	0.084	0.097	0.101	0.095	0.106	0.115	0.122	0.121
	48.60%	4.00	0.116	0.109	0.094	0.085	0.070	0.061	0.054	0.066	0.069	0.074	0.068	0.076	0.074	0.085	0.089
	47.99%	5.00	0.125	0.128	0.105	0.093	0.088	0.074	0.076	0.076	0.086	0.081	0.078	0.078	0.077	0.086	0.093
IA-2A	29.65%	(confirm visit) 0.00	0.218	0.248	0.269	0.285	0.288	0.310	0.323	0.314	0.321	0.323	0.327	0.319	0.307	0.300	0.305
	30.70%	0.25	0.165	0.201	0.231	0.253	0.266	0.306	0.315	0.310	0.329	0.329	0.345	0.335	0.321	0.316	0.319
	31.99%	0.50	0.104	0.154	0.193	0.227	0.253	0.294	0.321	0.312	0.332	0.338	0.339	0.338	0.331	0.325	0.326
	34.22%	1.00	*0.033*	0.093	0.142	0.182	0.217	0.261	0.297	0.296	0.314	0.321	0.337	0.337	0.324	0.322	0.324
	35.78%	1.50	*-0.001*	0.039	0.095	0.145	0.181	0.230	0.263	0.272	0.294	0.308	0.323	0.322	0.317	0.315	0.310
	36.85%	2.00	*-0.006*	*0.018*	0.073	0.131	0.169	0.220	0.258	0.272	0.294	0.315	0.324	0.324	0.325	0.324	0.324
	36.69%	2.50	*-0.021*	*0.007*	0.047	0.102	0.142	0.206	0.247	0.268	0.285	0.301	0.314	0.316	0.313	0.316	0.316
	36.07%	3.00	*-0.009*	*0.022*	0.047	0.106	0.153	0.210	0.249	0.271	0.293	0.304	0.326	0.330	0.329	0.327	0.326
	37.45%	4.00	*-0.024*	*0.010*	0.038	0.086	0.139	0.203	0.239	0.263	0.290	0.308	0.324	0.336	0.334	0.330	0.328
	36.51%	5.00	*-0.021*	*0.007*	0.044	0.090	0.108	0.175	0.214	0.235	0.263	0.283	0.303	0.308	0.312	0.307	0.308
IAA	56.09%	(confirm visit) 0.00	0.177	0.220	0.229	0.252	0.250	0.269	0.260	0.277	0.294	0.281	0.293	0.291	0.295	0.286	0.288
	54.28%	0.25	0.171	0.210	0.220	0.240	0.256	0.266	0.265	0.289	0.309	0.298	0.305	0.304	0.307	0.308	0.306
	49.63%	0.50	0.190	0.216	0.213	0.226	0.237	0.240	0.240	0.256	0.270	0.269	0.282	0.276	0.286	0.274	0.274
	43.41%	1.00	0.267	0.280	0.266	0.290	0.282	0.273	0.269	0.267	0.265	0.256	0.255	0.256	0.251	0.242	0.247
	41.09%	1.50	0.315	0.344	0.302	0.306	0.290	0.298	0.278	0.283	0.295	0.280	0.283	0.276	0.274	0.275	0.269
	38.97%	2.00	0.338	0.368	0.357	0.333	0.311	0.300	0.284	0.281	0.277	0.280	0.288	0.286	0.297	0.293	0.292
	37.07%	2.50	0.377	0.434	0.410	0.411	0.393	0.376	0.339	0.339	0.332	0.323	0.323	0.319	0.320	0.311	0.313
	36.54%	3.00	0.405	0.459	0.463	0.445	0.405	0.387	0.354	0.344	0.344	0.337	0.352	0.348	0.348	0.338	0.338
	33.36%	4.00	0.467	0.530	0.534	0.527	0.453	0.408	0.377	0.368	0.357	0.351	0.370	0.364	0.365	0.352	0.363
	32.65%	5.00	0.459	0.536	0.546	0.547	0.552	0.453	0.402	0.394	0.362	0.348	0.354	0.347	0.366	0.357	0.352

ESM Figure 8: Beta coefficients for the GADA, IA-2A, and IAA islet autoantibody levels at the third visit as a function of follow-up observation (T, ranging from 1 to 15 years along the horizontal axis) and interval from confirmatory visit to next test (W, ranging from 0.25 to 5 years along the vertical axis). The prediction time point ("Time 0") was the time of the third visit (confirmatory visit + W). The follow-up observation starts from the prediction time point. All prediction models used just 3 covariates: GADA, IA-2A, and IAA levels from the third visit. Statistically significant (p-value < 0.01) coefficients are shown in black text; values in gray italics text are not statistically significant. The percentage of subjects positive for each islet autoantibody at "Time 0" is shown under the "IAb Positivity" column.

		Follow-up observation - T (years)														
		**1**	**2**	**3**	**4**	**5**	**6**	**7**	**8**	**9**	**10**	**11**	**12**	**13**	**14**	**15**
**(confirm visit)**	**0.00**	0.8116	0.8211	0.7864	0.7779	0.7685	0.7686	0.7586	0.7572	0.7582	0.7571	0.7569	0.7516	0.7456	0.7390	0.7370
**Interval from confirm visit to next test - W (years)**	**0.25**	0.7922	0.8170	0.7836	0.7762	0.7735	0.7742	0.7674	0.7671	0.7706	0.7690	0.7698	0.7633	0.7559	0.7506	0.7496
	**0.50**	0.8155	0.8207	0.7843	0.7791	0.7743	0.7758	0.7732	0.7708	0.7737	0.7728	0.7748	0.7698	0.7632	0.7573	0.7535
	**1.00**	0.8455	0.8162	0.7796	0.7867	0.7818	0.7842	0.7780	0.7760	0.7779	0.7762	0.7783	0.7746	0.7680	0.7618	0.7618
	**1.50**	0.8657	0.8535	0.7803	0.7804	0.7708	0.7757	0.7705	0.7693	0.7727	0.7741	0.7737	0.7697	0.7650	0.7586	0.7558
	**2.00**	0.8714	0.8678	0.7902	0.7786	0.7716	0.7730	0.7706	0.7695	0.7693	0.7704	0.7746	0.7711	0.7718	0.7681	0.7626
	**2.50**	0.8751	0.8771	0.8030	0.7953	0.7846	0.7838	0.7761	0.7770	0.7754	0.7764	0.7790	0.7746	0.7720	0.7657	0.7630
	**3.00**	0.8843	0.8840	0.8444	0.8165	0.7978	0.7951	0.7846	0.7840	0.7836	0.7854	0.7893	0.7855	0.7838	0.7761	0.7740
	**4.00**	0.8902	0.8907	0.8537	0.8410	0.8064	0.7962	0.7860	0.7866	0.7867	0.7875	0.7911	0.7861	0.7825	0.7797	0.7743
	**5.00**	0.8885	0.8917	0.8582	0.8465	0.8379	0.8047	0.7891	0.7867	0.7828	0.7819	0.7867	0.7800	0.7763	0.7725	0.7610

ESM Table 1: Type 1 diabetes prediction performance (in IPCW concordance index) for different follow-up observations (T), ranging from T = 1 to 15 years, along the horizontal axis and different intervals from confirmatory visit to the next test (W), ranging from W = 0.25 to 5 years, along the vertical axis (W = 0 is the confirmatory visit). The prediction time point ("Time 0") was the time of the third visit (confirmatory visit + W). The follow-up observation starts from the prediction time point. All prediction models used just 3 covariates: GADA, IA-2A, and IAA levels from the third visit. IPCW indicates inverse probability censored weighting.
